# Perioperative blood transfusion is associated with a gene transcription profile characteristic of immunosuppression: a prospective cohort study

**DOI:** 10.1186/s13054-014-0541-x

**Published:** 2014-10-01

**Authors:** Paraskevi C Fragkou, Hew D Torrance, Rupert M Pearse, Gareth L Ackland, John R Prowle, Helen C Owen, Charles J Hinds, Michael J O’Dwyer

**Affiliations:** Adult Critical Care Unit, Royal London Hospital, Barts Health NHS Trust, Whitechapel Road, London, E1 1BB UK; Centre for Translational Medicine and Therapeutics, William Harvey Research Institute, Barts and the London School of Medicine and Dentistry, Queen Mary University of London, Charterhouse Square, London, EC1M 6BQ UK; Department of Medicine, Wolfson Institute for Biomedical Research, University College London, Cruciform Building, Gower Street, London, WC1E 6BT UK

## Abstract

**Introduction:**

Blood transfusion in the perioperative period has frequently been associated with an excess of nosocomial infections. Whilst transfused whole blood induces specific host immune alteration that may predispose to nosocomial infections, the immunomodulating properties associated with leukodepleted blood remain incompletely understood. In this study, we explore the hypothesis that the transfusion of leukodepleted allogeneic blood during or following major gastrointestinal surgery is associated with an immunosuppressed phenotype, which may in turn predispose to postoperative infectious complications.

**Methods:**

Patients aged over 45 years undergoing scheduled inpatient major gastrointestinal surgery were recruited. Gene expression profiles of specific inflammatory genes were assayed from blood collected preoperatively, at 24 and at 48 hours after surgery. Genes were selected based on their ability to represent specific immune pathways. Gene expression was quantified using quantitative real-time polymerase chain reaction (qRT-PCR) to measure messenger RNA (mRNA) levels. Postoperative infections were documented using predefined criteria.

**Results:**

One hundred and nineteen patients were recruited. Fifteen (13%) patients required blood transfusion within 24 hours of surgery, 44 (37%) patients developed infections and 3 (2%) patients died prior to discharge. Patients receiving a blood transfusion were more likely to develop postoperative infections (*P* =0.02) and to have lower tumour necrosis factor alpha (TNFα), interleukin (IL)-12, IL-23 and RAR-related orphan receptor gamma T (RORγt) gene expression in the postoperative period (*P* <0.05). The TNFα/IL-10 mRNA ratio at 24 hours (*P* =0.0006) and at 48 hours (*P* =0.01) was lower in patients receiving a blood transfusion over this period. Multivariable analysis confirmed that these observations were independent of the severity of the surgical insult.

**Conclusions:**

An association between an immunosuppressive pattern of gene expression and blood transfusion following major elective gastrointestinal surgery is described. This gene expression profile includes a reduction in the activity of innate immunity and T helper cell type 1 (T_h_1) and T helper cell type 17 (T_h_17) pathways in those patients receiving a blood transfusion. Blood transfusion was also associated with an excess of infectious complications in this cohort. A mechanistic link is suggested but not proven.

**Electronic supplementary material:**

The online version of this article (doi:10.1186/s13054-014-0541-x) contains supplementary material, which is available to authorized users.

## Introduction

The immunomodulating qualities of allogenic blood transfusion have long been appreciated and have even been exploited to prevent renal allograft rejection in the era prior to the development of effective immunosuppressant drugs [1]. The unintended clinical consequences of immune modulation by allogenic blood in the perioperative period include an increased susceptibility to infectious complications and cancer recurrence [2,3]. As inpatient non-cardiac surgery has recently been associated with much higher than anticipated mortality rates, it is now imperative that all potentially avoidable causes of excess morbidity in this population are investigated and addressed [4]. Whilst preoperative anaemia is associated with a doubling of inhospital mortality, it remains unclear whether the anaemia itself or its treatment with allogeneic blood is responsible [5]. Furthermore, perioperative blood transfusions are not universally associated with an increase in complications particularly when patients receiving leukodepleted blood are included [2,6]. Also lacking from the current literature is an analysis of alterations in key inflammatory pathways associated with the transfusion of leukodepleted blood following major elective surgery, although our group has recently described an association between blood transfusion following severe traumatic injury, an anti-inflammatory pattern of gene expression and an excess of infectious complications [7].

It has been repeatedly demonstrated that messenger RNA (mRNA), assayed using real-time polymerase chain reaction (RT-PCR), can be used to accurately quantify patterns of gene expression [8-10]. Furthermore, careful selection of key regulatory and effector cytokines in conjunction with transcription factors specific to T cell subtypes allow inferences to be made as to the activity of specific inflammatory pathways related to T-helper (T_h_) cell development [11]. For example, an analysis that combines gene expression data relating to the key T_h_1-promoting cytokine, interleukin (IL)-12, the main T_h_1 effector cytokine, interferon gamma (IFNγ) and the T_h_1-specific transcription factor T-bet may be descriptive of the general activity of the T_h_1 pathway. A similar approach can aid in the description of T-helper cell type 2 (T_h_2), T_h_17 and T regulatory (T_reg_) cell pathways. This methodology has been previously utilised to predict perioperative infectious complications based on alterations in T cell pathways [10].

In this study, we explore the hypothesis that blood transfusion during and following major elective gastrointestinal surgery is associated with an immunosuppressive pattern of gene expression, which may enhance susceptibility to later infectious complications.

## Methods

This prospective cohort study was conducted at The Royal London Hospital, London UK and University College Hospital, London between June 2012 and November 2013. Ethical approval was granted by the North Wales Ethics Committee (Reference: 10/WNo03/25) and this research was conducted in compliance with the Helsinki Declaration. Each patient on a weekday elective operating list was screened. Eligible patients were then approached for written informed consent. Entry into the study did not influence the patient’s clinical treatment course in any way. In our institution, scheduled postoperative intensive care unit (ICU) admission is mandated for specific surgical procedures. Based on an individual patient’s co-morbidities the surgical or anaesthesia teams may also request postoperative ICU treatment. All patients received perioperative prophylactic antibiotic treatment as outlined in Additional file 1.

### Patient selection

Patients aged over 45 years undergoing scheduled surgery involving the gastrointestinal tract, requiring a general anaesthetic and at least an overnight hospital stay were considered eligible for inclusion to this study. Exclusion criteria were emergency surgery and surgery that also involved access to the thoracic cavity.

### Data collection

Data were collected on each patient until hospital discharge and included information on co-morbidities, American Society of Anesthesiology (ASA) physical status classification [12] indication for surgery, cancer diagnosis, duration of the procedure, planned postoperative ICU admission and inhospital mortality. Preoperative immunosuppression was defined as the administration of chemotherapeutic agents and/or radiotherapy within the six months preceding surgery. The timing of any blood products administered was recorded for each patient during the first 24 hours. All packed red blood cells (PRBC) administered were leukocyte depleted prior to storage, included a standard sodium chloride, adenine, glucose, mannitol (SAGM) additive solution and had a maximum age of 35 days. Patients were examined daily by the clinical team for the presence of infection, which was then recorded by the research team following discussions with ICU physicians, surgeons or microbiologists where necessary. Definitions of infection were agreed prospectively by the investigators and were based on the Center for Disease Control and Prevention definitions and graded using the Clavien-Dindo classification (see Additional file 2) [13].

### Blood sampling and laboratory techniques

Blood samples were taken immediately before induction of anaesthesia (0 hours), at 24 and 48 hours following the operation. At each time point blood was collected in a PAXGene™ blood RNA tube (PreAnalytix, Hilden, Germany). Total RNA was extracted using PAXGene™ blood RNA kit (PreAnalytix). Samples were analysed for their integrity and reverse transcribed to complementary DNA (cDNA) [11]. Genes were selected to reflect key elements of specific immune pathways (Table 1). Gene expression was quantified using quantitative real-time polymerase chain reaction (qRT-PCR). qRT-PCRs were performed using Taqman assays (Applied Biosystems, Foster City, CA, USA), which spanned the final two exons of the most common isoform of each gene, and were carried out on a 7900HT, Life Tech (Applied Biosystems, Foster City, CA, USA) as previously described [7]. Each sample was assayed in triplicate. Reference genes (β2 microglobulin (*B2M*) and ubiquitin C (*UBC*)) were selected empirically from a panel of six [14]. Relative quantification was calculated using the standard delta delta methodology [15]. Results were expressed as a normalized ratio of candidate gene/reference gene.

**Table 1:** **Selected cytokines and transcription factors and their related pathways**

**Gene**	**Immune pathway**	**Contributes to anti (-) or pro (+) inflammatory phenotype** ^**a**^
TNFα (*TNF*)^b^	Common end product of many innate and adaptive immune pathways	**+**
IFNγ (*IFNG*)^b^	T_h_1 effector cytokine	**+**
IL-12 (*IL12A*)^b^	Promotes differentiation to T_h_1 effector cells	**+**
T-bet (*TBX21*)^b^	Transcription factor utilised by T_h_1 cells	**+**
IL-23 (*IL23A*)^b^	Promotes differentiation to T_h_17 phenotype	**+**
IL-27 (*IL27*)^b^	Inhibits differentiation to T_h_17 phenotype	**-**
RORγT (*RORC*)^b^	Transcription factor utilised by T_h_17 cells	**+**
IL-10 (*IL10*)^b^	Anti-inflammatory cytokine produced by many T cell subtypes and some macrophages	**-**
Foxp3 (*FOXP3*)^b^	Transcription factor utilised by naturally occurring CD4^+^ CD25^+^ T_reg_ cells	**-**
GATA3 (*GATA3*)^b^	Transcription factor utilised by T_h_2 cells	**-**

^a^Cytokines and transcription factors will have diverse actions under different conditions and the descriptions above are primarily for illustrative purposes and are not exhaustive; ^b^denotes official gene name as described by the HUGO Gene Nomenclature Committee (HGNC). Foxp3, forkhead box P3; GATA3, GATA-binding protein 3; IFNγ, interferon gamma; IL-12, interleukin 12; IL-23, interleukin 23; IL-27, interleukin 27; RORγt, RAR-related orphan receptor gamma T; T_h_1, T-helper cell type 1; T_h_17, T-helper cell type 17; T_h_2, T-helper cell type 2; TNFɑ, tumour necrosis factor alpha; T_reg_, T regulatory cell.

### Statistical analysis

Results are expressed as median and interquartile range (IQR) or odds ratios (OR) with 95% confidence intervals (CI). All statistical tests are two-sided and a *P* value of *P* <0.05 was considered significant. Differences in categorical variables were calculated using a chi-squared or Fisher’s exact test as appropriate. The Kruskal-Wallis test was utilised for continuous variables.

Multivariable linear regression models assessed whether gene expression was independently associated with blood transfusion. A model was constructed in each case where gene expression was demonstrated to be associated with blood transfusion on a univariate analysis. Gene expression was then selected as the dependent variable following log transformation of the mRNA levels. A univariate analysis then assessed the relationship between gene expression and age, sex, duration of operation, ASA physical status classification, cancer diagnosis and preoperative immunosuppression. If this univariate analysis demonstrated a *P* value <0.1 then that variable was added to the above model as an independent variable. Backwards elimination on non-significant variables was performed when appropriate (see Additional file 3).

A multivariable logistical regression analysis assessed whether infectious complications were independently associated with blood transfusion. The occurrence of infectious complications was the dependent variable and the threshold for inclusion of other variables was again *P* <0.1, with variables selected from Table 2 (see Additional file 4).

**Table 2:** **Characteristics of patients developing infections and those remaining infection-free following scheduled abdominal surgery**

	**Infection, n =44 (37%)**	**Infection-free, n =75 (63%)**	***P*** **value**
Age (years)	66 (59-75)	64 (56-71)	0.19
Male sex (%)	61	63	>0.99
Diabetes (%)	18	16	0.80
Current smokers (%)	23	19	0.64
Smoking history (%)	48	57	0.34
Cancer diagnosis (%)	55	71	0.10
Preoperative immunosuppression (%)	14	14	>0.99
Duration of operation (minutes)	243 (176-313)	195 (142-295)	0.06
Endoscopic surgery (%)	18	32	0.13
Planned postoperative intensive care unit admission (%)	77	66	0.22
ASA grade 3 or 4 (%)	30	31	>0.99
**By surgical specialty n (%)**			
General surgery	4 (44)	5 (55)	
Upper gastrointestinal	9 (33)	18 (67)	
Colorectal	18 (37)	31 (63)	
HPB	11 (37)	19 (63)	
HPB + colorectal	1 (33)	2 (67)	
General surgery + colorectal	1 (100)	0 (0)	0.84^a^
Intraoperative blood transfusion (%)	14	5	0.17
Blood transfusion in the first 24 hours (%)	23	7	0.02
Inhospital death n (%)	1 (2%)	2 (2.5%)	>0.99

Data are described as medians with interquartile range or numbers with percentages in parenthesis. mRNA levels are expressed as a relative quantification ratio between the candidate and the reference genes. ^a^Represents a Fisher’s exact test incorporating all surgical specialties. ASA, American Society of Anesthesiology; HPB, Hepato-Pancreato-Biliary.

Data analysis was performed using the JMP (version 11) statistical software package (SAS Institute Inc., Cary, NC, USA).

## Results

### Patients

A total of 119 patients (mean age 65, range 57 to 72, 62% male) undergoing elective major abdominal surgery were included in this study. Fifteen (13%) patients received a blood transfusion. Forty-four (37%) patients developed a postoperative infection. Three (2.5%) patients died prior to hospital discharge. Patient characteristics are shown in Tables 2 and 3.

**Table 3:** **Characteristics of patients requiring perioperative blood transfusion following scheduled abdominal surgery**

	**Transfused, n =15 (13%)**	**Not transfused, n =103 (87%)**	***P*** **value**
Age (years)	77 (72-81)	63 (56-70)	0.0002
Male sex (%)	60	62	1.0
Diabetes (%)	20	17	0.72
Current smokers (%)	0	23	0.04
Smoking history (%)	27	58	0.02
Cancer diagnosis (%)	93	60	0.02
Preoperative immunosuppression (%)	29	12	0.10
Duration of operation (minutes)	240 (150-400)	212 (145-296)	0.26
Endoscopic surgery (%)	13	29	0.35
Planned postoperative intensive care unit admission (%)	87	68	0.22
ASA grade 3 or 4 (%)	50	28	0.18
**By surgical specialty n (%)**			
General surgery	0 (0)	8 (7)	
Upper gastrointestinal	4 (27)	23 (22)	
Colorectal	5 (33)	44 (43)	
HPB	5 (33)	25 (24)	
HPB + colorectal	1 (7)	2 (2)	
General surgery + colorectal	0 (0)	1 (1)	0.65^a^
Postoperative infections n (%)	10 (66%)	34 (33%)	0.02
Inhospital death n (%)	2 (13%)	1 (1%)	0.04

Data are described as medians with interquartile range or numbers with percentages in parenthesis. mRNA levels are expressed as a relative quantification ratio between the candidate and the reference genes. ^a^Represents a Fisher’s exact test incorporating all surgical specialties. ASA, American Society of Anesthesiology; HPB, Hepato-Pancreato-Biliary.

### Blood transfusion

**Figure 1:**
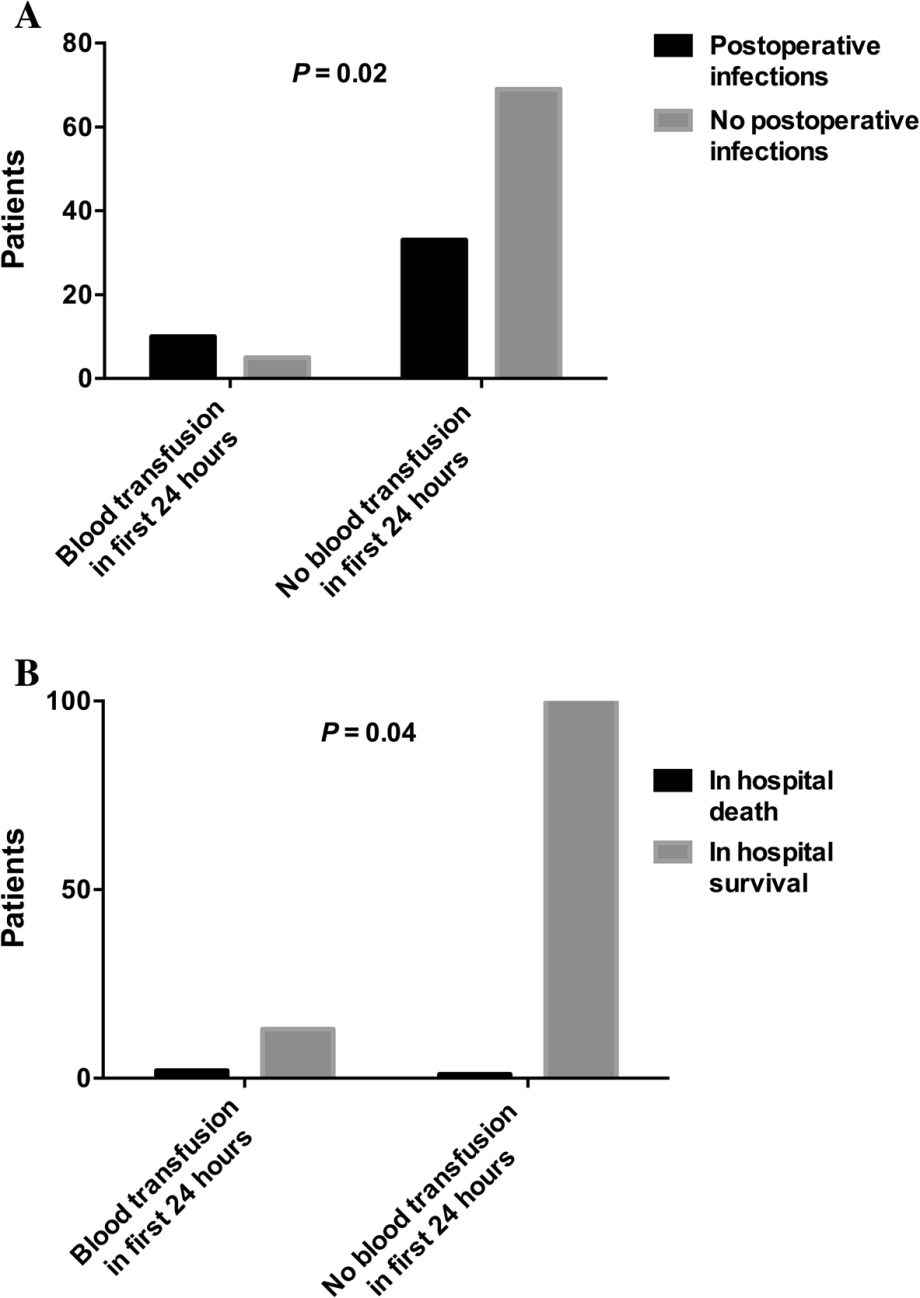
**Perioperative blood transfusion, infectious complications and survival.** Graph **A** represents the proportions of patients developing postoperative infectious complications amongst those patients receiving a blood transfusion in the first 24 hours and in those patients not receiving a blood transfusion during this time period. Graph **B** represents the proportions of patients either dying in hospital or surviving to hospital discharge amongst those patients that received a blood transfusion in the first 24 hours (2 patients died out of a total of 15) and in those patients that did not receive a blood transfusion during this time period (1 patient died out of a total of 104).

In total 15 (13%) patients were transfused over the 24 hours following the commencement of surgery. The median number of units of PRBCs transfused in this group was 2 (IQR 1 to 2). Ten of these 15 patients received an intraoperative transfusion, 8 of these 15 received their transfusion in the postoperative period and, therefore, 3 of the 15 patients received a transfusion both intraoperatively and postoperatively. No other blood product was transfused during this time period. Older patients (*P* =0.0002) and those with a diagnosis of cancer (*P* =0.02) were more likely to receive PRBC transfusions. Smokers were less likely to receive a blood transfusion (*P* =0.04). The requirement for blood transfusion was not related to the duration of the operation, whether the procedure was endoscopic or open or the ASA grade (Table 3). Patients receiving PRBC transfusions were more likely to develop postoperative infections (OR 5.5 (1.3 to 12.8); *P* =0.02 (Fisher’s exact test), Figure 1A) and were more likely to die inhospital (OR 15.7 (1.3 to 185.3); *P* =0.04 (Fisher’s exact test), Figure 1B).

### Blood transfusion and gene expression

**Figure 2:**
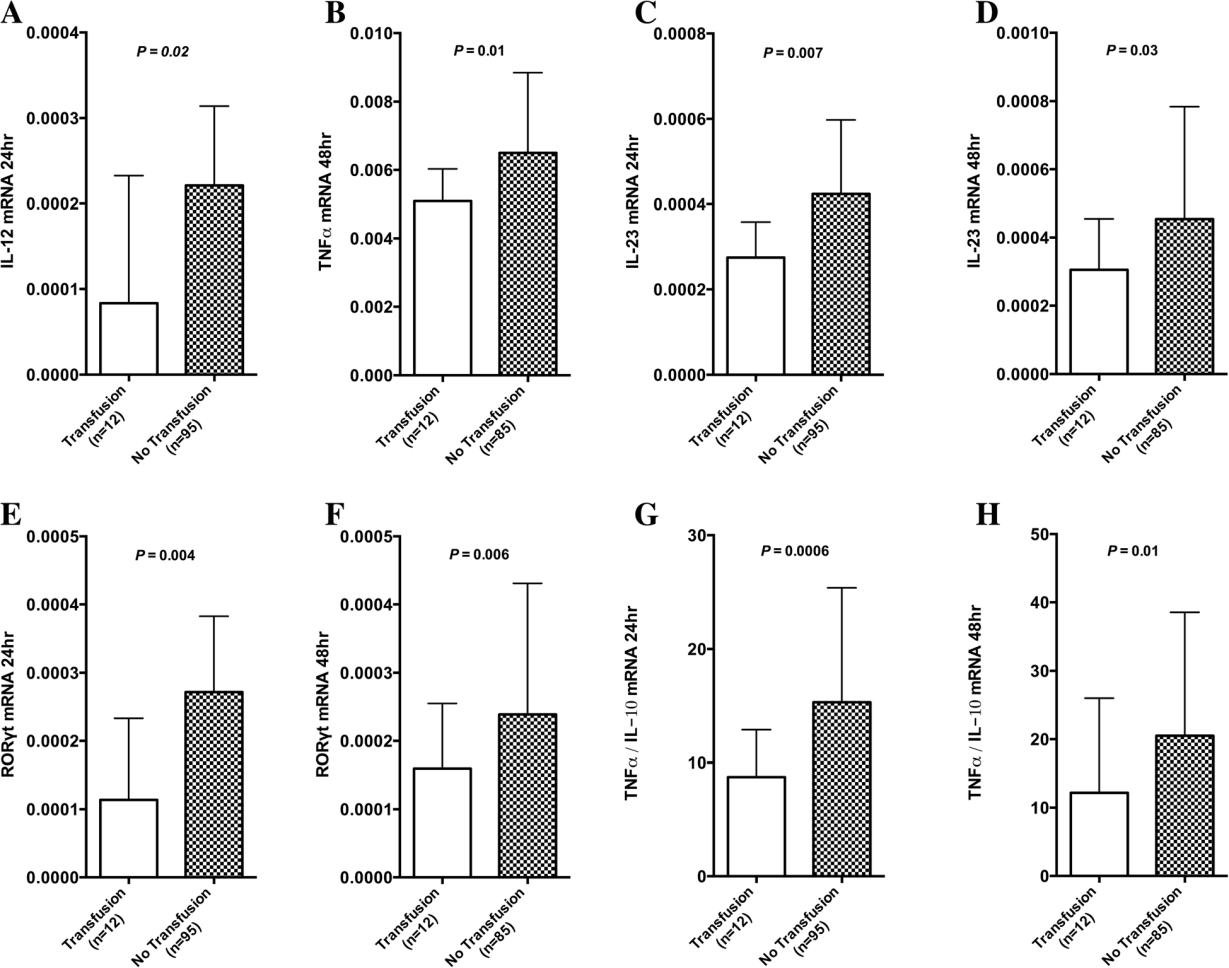
**Perioperative blood transfusion and gene expression: Graphs A-H represent the mRNA levels of IL-12 at 24 hours, TNFα at 48 hours, IL-23 at 24 and 48 hours, RORγt at 24 and 48 hours and the ratio of TNFα/IL-10 at 24 and 48 hours following scheduled abdominal surgery in those patients that received a transfusion prior to this time point and in those patients that did not.** Graphs represent median and 75th percentile. Results are expressed as a relative quantification ratio between the candidate and the reference genes. IL-10, interleukin 10; IL-12, interleukin 12; IL-23, interleukin 23; mRNA, messenger RNA; RORγt, RAR-related orphan receptor gamma T; TNFɑ, tumour necrosis factor alpha.

On univariate analysis, IL-12 mRNA levels at 24 hours (*P* =0.02) and tumour necrosis factor alpha (TNFα) mRNA levels at 48 hours (*P* =0.01) were lower in those who received a blood transfusion in the first 24 hours postoperatively. IL-23 mRNA levels at 24 hours (*P* =0.007) and at 48 hours (*P* =0.03) and RAR-related orphan receptor gamma T (RORγt) mRNA levels at 24 hours (*P* =0.004) and at 48 hours (*P* =0.006) were lower in those receiving a blood transfusion over the first 24 hours postoperatively. The TNFα/IL-10 mRNA ratio at 24 hours (*P* =0.0006) and at 48 hours (*P* =0.01) was lower in patients receiving blood transfusion over this period (Figure 2A-H).

Forkhead box P3 (Foxp3) mRNA levels at 24 hours (*P* =0.004) and at 48 hours (*P* =0.02) and GATA-binding protein 3 (GATA3) mRNA levels at 24 hours (*P* =0.02) were lower in those who received blood transfusion in the initial 24 hours postoperatively. IL-10, IFNγ, T-bet and IL-27 mRNA levels were unrelated to blood transfusion.

The number of units of blood transfused was unrelated to gene expression.

### Mean age of the transfused blood and gene expression

The age of the blood was calculated as the number of days from collection from a blood donor to transfusion. The mean age of the transfused blood was 23 days (standard deviation 5.9 days, range 11 to 33 days). No association was detected between the mean age of the units of blood transfused and gene expression for any of the selected genes.

### Postoperative infections

Forty-four (37%) patients developed postoperative infections as an inpatient a median of 9 (IQR 5 to 11) days following the operation. The sites of infection and isolated organisms are shown in Table 4. Patients developing infections stayed longer in hospital (7 (5 to 10) vs. 14 (8 to 19) days, *P* <0.0001). With the exception of perioperative blood transfusion, a range of demographic and clinical data did not adequately distinguish between those who did and did not develop infection (Table 2).

**Table 4:** **A description of the infectious complications in the postoperative period**

**Infection site**	**Number of episodes**	**Organisms**	**Median time to development of infection (days)**
**Bloodstream infection**	11	*Escherichia coli* (4)	17 (6-28)
		ESBL *E. coli* (1)	
		*E. coli + Enterococcus faecium* (1)	
		*E. faecium* (1)	
		*Klebsiella pneumoniae* (1)	
		*Staphylococcus aureus* (1)	
		*Pseudomonas aeruginosa* (2)	
**Pneumonia**	9	Culture negative (5)	7 (4-16)
		*Candida glabrata* (1)	
		*K. pneumoniae* (1)	
		Organism of the coliform group (1)	
		*Stenotrophomonas maltophilia* (1)	
**Wound infection**	24	Culture negative (11)	10 (4-14)
		*Bacteroides fragilis* (1)	
		*E. coli* (4),	
		ESBL *E. coli* (1)	
		Mixed anaerobes (1)	
		Organism of the coliform group (2)	
		Organism of the coliform group + *E. faecalis* (1)	
		Organism of the coliform group + MRSA (1)	
		Organism of the coliform group + *S. aureus* (1)	
		*P. aeruginosa* (1)	
**Urinary tract infection**	15	*Candida albicans* (1)	8 (6-12)
		Culture negative (2)	
		*E. coli* (6)	
		ESBL *E. coli* (1)	
		Mixed growth (2)	
		Organism of the coliform group (2)	
		*P. aeruginosa* (1)	
**Intra-abdominal infection**	17	Culture negative (6)	9 (7-14)
		*E. coli* (2)	
		*E. coli* + *E. faecium* (1)	
		ESBL *E. coli* + VRE (1)	
		*E. faecalis* (1)	
		*E. faecium* + *Candida albicans* (1)	
		*C. albicans* (1)	
		*Enterobacter cloacae* (1)	
		*E. cloacae* + Organism of the coliform group (1)	
		*E. aerogenes* + *P. aeruginosa* (1)	
		VRE (1)	
**Intravascular catheter- related infection**	2	*K. pneumoniae* (1)	15 (3-27)
		Organism of the coliform group (1)	
**Skin infection**	1	Varicella zoster virus (VZV)	30

Data refer to the number of episodes of infection from a particular site. Some patients may have more than one episode of infection. The number in parenthesis in the ‘organisms’ column the number of episodes of infection attributable to that organism. Data are described as median and interquartile range. ESBL, extended-spectrum beta-lactamase; MRSA, methicillin-resistant *Staphylococcus aureus*; VRE, vancomycin-resistant *Enterococcus*.

### Inhospital mortality

Three (2.5%) patients died in hospital. Age, diabetes, smoking history, ASA, length of operation, cancer diagnosis, planned ICU admission, preoperative immunosuppression or postoperative infections were not associated with inhospital mortality. The requirement for blood transfusion in the first 24 hours postoperatively was associated with a greater mortality (*P* =0.04; Figure 1B).

### Multivariable analysis

Selection of variables to be included in the multivariate analysis is described in the Methods section. IL-12, RORγt, TNFα/IL-10 and Foxp3 mRNA levels at 24 hours along with RORγt, TNFα/IL-10 and Foxp3 mRNA levels at 48 hours were independently associated with transfusion in the first 24 hours (see Additional file 3). There was no independent association between GATA3 and IL-23 mRNA levels at 24 hours and between IL-23 and TNFα mRNA levels at 48 hours and blood transfusion within the first 24 hours.

There was an independent association between the occurrence of infectious complications and the requirement for blood transfusion in the first 24 hours postoperatively (see Additional file 4).

### Differential leucocyte count and gene expression

In order to assess for an association between perioperative variations in white blood cell subpopulations and gene expression, changes in neutrophil, lymphocyte, monocyte and eosinophil counts over the initial 24 hours were compared with changes in individual gene expression over the same period (see Additional file 5). Thirty-six comparisons were generated. Of these, just two attained a level of statistical significance with *P* <0.05 before correcting for multiple comparisons. An association was noted between increasing IL-10 mRNA levels and increasing neutrophil counts (r^2^ = 0.06, *P* =0.01) and between decreasing lymphocyte counts and increasing IL-10 mRNA levels (r^2^ = 0.05, *P* =0.02).

## Discussion

In this study we determined levels of mRNA for a panel of interlinked cytokines and related transcription factors and demonstrated that those patients receiving a perioperative blood transfusion following major gastrointestinal surgery displayed a pattern of gene expression consistent with greater immunosuppression when compared with a cohort not receiving a blood transfusion. This observation was independent of variables descriptive of the extent of surgical trauma. The gene expression data presented suggests that blood transfusion in this setting may be associated with specific immune defects in innate immunity and in T_h_1 and T_h_17 pathways. These patients also had an excess of postoperative infectious complications when compared with patients that did not receive a blood transfusion; an observation that was again independent of the duration of the surgical procedure.

The genes selected as candidates in this study are well described as surrogate measures of the specific immune pathways outlined in Table 1 [10,11,16]. Furthermore, the construction of a ratio of gene expression that incorporates the prototypical proinflammatory cytokine, TNFα, and the archetypal anti-inflammatory cytokine, IL-10, permits the concurrent assessment of both major facets of the immune response and has previously been utilised to predict postoperative outcome [17]. Whilst the observed reductions in TNFα and IL-12 gene expression are indicative of impaired innate immunity and T_h_1 pathways respectively and the reduction in IL-23 and RORγt is indicative of reduced T_h_17 activity, these data should not be interpreted as being conclusive. Flow cytometry experiments would be necessary to confirm these initial data but present significant logistical barriers and are ideally not performed on stored samples [18]. The T_h_1 pathway is an essential link between innate and adaptive immunity and T_h_1 effector cells are particularly important for effective bactericidal activity against intracellular pathogens [19]. T_h_17 cells are a branch of the adaptive immune system and appear to deal primarily with organisms inadequately subdued by T_h_1 or T_h_2 immunity and that seem to require a very robust inflammatory response [20]. It is biologically plausible that an acquired immune deficit involving these pathways could lead to an increased susceptibility to opportunistic infections with the pathogens encountered in the perioperative period.

Although numerous small studies have suggested that perioperative allogenic blood transfusion may induce immune modulations that create an environment that promotes both microbial growth and tumorigenesis their conclusions are limited by small cohort size, *in vitro* and *ex vivo* assays and insensitive protein assays [3,21-26]. In addition, these studies largely predate the routine leukodepletion of transfused blood and suggest a causal relationship between immune modulation and the infusion of allogeneic leukocytes; a biological effect that could be ameliorated by pre-storage leukodepletion [22]. It cannot therefore be inferred that a similar immunological response will be observed following exposure to a leukodepleted product. In contrast, the packed red cells transfused in the study reported here were universally leukodepleted. Furthermore, the use of qRT-PCR in this study to assay gene expression allows for a validated ultrasensitive technique to assess immune pathways in the *in vivo* state [8-11]. The results we present suggest that the immunosuppression observed following perioperative transfusion may not be solely dependent on the presence of allogeneic leukocytes in the transfused blood. However, despite a 3 log reduction in leukocyte numbers achieved following current filtration techniques some leukocytes do persist and these retain the ability to influence host immune responses [22].

Recently, using a similar methodology we described an excess of infectious complications amongst severely injured trauma patients receiving leukodepleted blood transfusion in conjunction with a gene expression profile characterised by specific deficits in T_h_1 and T_h_17 pathways [7]. The similarities between these results are notable given that there were important differences in the patient populations. The trauma cohort were younger (median age 41), had a higher incidence of early blood transfusion (64%) and received greater volumes of PRBCs within the initial 24 hours (median 4 units) in conjunction with additional blood products. In addition, the trauma cohort are likely to have endured a greater physiological insult given a higher mortality rate (19%) and also a greater incidence of nosocomial infections (63%). Clearly, patients that receive a blood transfusion outside of the context of a randomised controlled trial will differ from those that do not require transfusion and it is important to attempt to control for these factors. In the previously reported trauma cohort the described observations were independent of the severity of injury and degree of shock at presentation [7]. Similarly, in this perioperative cohort, although on univariate analysis there was a trend towards longer and therefore potentially more complicated surgery being associated with postoperative infections and to a lesser degree with blood transfusion, a multivariable analysis confirmed that the association between blood transfusion and both infectious complications and gene expression patterns were independent of the duration of surgery. Although we describe an association between blood transfusion, an immunosuppressive pattern of gene expression and excess infectious complications, it may be that, despite adjustment for known confounders, the need for blood transfusion is a surrogate marker of severity of surgical stress or an unmeasured premorbid condition and thus a causative role in postoperative immune phenotype is suggested but not proven. However, the consistency of these data from two distinct patient populations, post major gastrointestinal surgery patients and trauma patients, strengthens our underlying hypothesis.

The association between blood transfusion and infectious complications in the perioperative period has been well described previously [2,3,27]. This association appears to be consistent amongst cohorts that received either whole blood or leukodepleted blood. The magnitude of the effect size is potentially quite substantial with one recent estimate quantifying the increased risk of infection at 29% for each unit of blood transfused [27]. That we were able to describe a similar association between blood transfusion and nosocomial infection in a smaller cohort of patients may be related to this effect size, in addition to the selection of an at-risk population using patient age, gastrointestinal surgery under general anaesthesia and the requirement for inpatient treatment as inclusion criteria. We did not, however, record blood transfusions that were administered following the initial 24 hours postoperatively. The postoperative infection rates we report are consistent with similar patient populations [28]. This study also described an association between blood transfusion and mortality. However, whilst the mortality rate is consistent with similar populations [28], with only three deaths occurring in the cohort as a whole, no conclusions regarding causation should be inferred from this association.

It is unsurprising that we did not observe an association between increasing age of the transfused blood and either perioperative infectious complications or patterns of gene expression as this study was underpowered to detect such an association. There is much current debate surrounding the potential pathogenicity associated with blood stored for excessive periods prior to transfusion. In the perioperative period, blood stored for longer periods has been linked to an excess of infectious complications [29-31]. The results of a randomised controlled trial comparing blood stored for under a week with older blood in intensive care patients are awaited and will provide important and relevant data [32].

## Conclusions

Our data suggest that the transfusion of leukodepleted allogeneic blood during or immediately after major gastrointestinal surgery is associated with patterns of gene expression consistent with immunosuppression and specific deficiencies in innate immunity and T_h_1 and T_h_17 proinflammatory immune pathways. A mechanistic link between allogenic perioperative blood transfusion, defects in essential bactericidal pathways and an excess of nosocomial infections is suggested but not proven. Further research is necessary in order to prove causation.

## Key messages

- Perioperative blood transfusion is associated with an excess of nosocomial infections.
- Perioperative blood transfusion is associated with a specific gene expression profile indicative of immune suppression that could predispose patients to nosocomial infections.

## Additional files

Additional file 1:
**Antibiotic prophylaxis for GI surgery in adults.** Institutional guidelines for perioperative antibiotic prophylaxis in adult patients undergoing gastrointestinal surgery. Additional file 2:
**Criteria used for defining the sites of infection.** Criteria used for defining the infection sites based on the Center for Disease Control and Prevention definitions [13]. Additional file 3:
**Multivariable linear regression analysis for prediction of gene expression.** Multivariable linear regression analysis of gene expression using requirement for blood transfusion, diagnosis of cancer, duration of the surgical procedure, age, ASA class and the presence of preoperative immunosuppression transfusion as potential independent variables. Selection of variables is described in the Methods section. Additional file 4:
**Multivariable logistical regression analysis for postoperative infections.** Multivariable logistical regression analysis of postoperative infections using cancer diagnosis, duration of surgery and transfusion with the first 24 hours postoperatively as independent variables. Selection of variables is described in the Methods section. Additional file 5:
**A comparison of changes in leucocyte subpopulations with changes in candidate gene expression.** Changes in leucocyte subpopulations over the perioperative period are expressed by calculating a ratio of the cell count at 24 hours to the preoperative cell count. The same calculation was used to assess the change in gene expression over the same period.

## Abbreviations

ASA: American Society of Anesthesiology
B2M: β2 microglobulin (*B2M*)
cDNA: complementary DNA
Ct: cycle threshold
Foxp3: forkhead box P3 (*FOXP3*)
GATA3: GATA-binding protein 3 (*GATA3*)
ICU: intensive care unit
IFNγ: interferon gamma (*IFNG*)
IL: interleukin
IL-10: interleukin 10 (*IL10*)
IL-12: interleukin 12 (*IL12A*)
IL-23: interleukin 23 (*IL23A*)
IL-27: interleukin 27 (*IL27*)
IQR: interquartile range
MHC: major histocompatibility complex
mRNA: messenger RNA
PRBC: packed red blood cells
qRT-PCR: quantitative real-time polymerase chain reaction
RORγt: RAR-related orphan receptor gamma T (*RORC*)
RT-PCR: real-time polymerase chain reaction
SAGM: sodium chloride, adenine, glucose, mannitol
T_h_: T-helper cell
T_h_1: T-helper cell type 1
T_h_17: T-helper cell type 17
T_h_2: T-helper cell type 2
TNFɑ: tumour necrosis factor alpha (*TNF*)
T_reg_: T regulatory cell
UBC: ubiquitin C (*UBC*)
