# Supplementary material for: Perioperative blood transfusion is associated with a gene transcription profile characteristic of immunosuppression: a prospective cohort study
Source: Crit Care. 2014 Oct 1;18(5):541. doi: 10.1186/s13054-014-0541-x (PMC4201915; doi:10.1186/s13054-014-0541-x)
Supplement: Additional file 1: — Antibiotic prophylaxis for GI surgery in adults. Institutional guidelines for perioperative antibiotic prophylaxis in adult patients undergoing gastrointestinal surgery. [file 13054_2014_541_MOESM1_ESM.pdf]

Antibiotic prophylaxis for GI surgery in Adults

- Colorectal surgery
- Appendicectomy
- Upper gastrointestinal surgery
- Open biliary surgery
- Laparoscopic procedures in high-risk patients
- Pancreatic and liver surgery
- Open or laparoscopic surgery with mesh (eg gastric band / rectopexy) – consider antibiotic prophylaxis in high-risk patients
- Hernia repair with or without mesh – SIGN guidance does not recommend antibiotic prophylaxis

Antibiotic prophylaxis is a commonly used method of reducing the incidence and impact of surgical site infections in a wide range of procedures. **Patients already on antibiotic treatment for intra-abdominal infection DO NOT require additional prophylaxis.**

Patients with current or previous infection or colonisation with resistant organisms may require alternative prophylaxis; please contact Microbiology to discuss.

A single (STAT) dose of antibiotic prophylaxis should be written on the STAT side of the inpatient chart. This should be administered at induction of anaesthesia.
In prolonged procedures (>4 hours) or where there is significant blood loss a second dose may be considered.

Cefuroxime IV 1.5g

PLUS

Metronidazole IV 500mg

For biliary surgery in the presence of stents, or pancreaticoduodenectomy, **ADD**

Gentamicin IV 2mg/kg, maximum dose 160mg

Cefuroxime will be restricted to theatres, and should **NOT** be continued for treatment of infection.

Alternative for patients with Type 1 penicillin allergy:

Gentamicin IV 2mg/kg, maximum dose 160mg

PLUS

Metronidazole IV 500mg

MRSA status MUST be checked prior to surgery.
For patients known to be currently or previously MRSA positive, **ADD to the above prophylaxis:**

Teicoplanin IV 800mg STAT

Written by: Dr Amin (Consultant Microbiologist), June 2013
Agreed by: Antimicrobial Review Group
Review: May 2015

If there is pus found in the abdomen, an acutely inflamed appendix, or bowel perforation has occurred, institute a therapeutic course of antibiotics **as per treatment guidelines** for intra-abdominal sepsis.

References:

1. National SIGN guidelines on antibiotic prophylaxis in surgery (2008). http://www.sign.ac.uk/pdf/sign104.pdf
2. *Antibacterial prophylaxis in surgery: 1 – Gastrointestinal and biliary surgery*. DTB 2003;41:83-86

Written by: Dr Amin (Consultant Microbiologist), June 2013
Agreed by: Antimicrobial Review Group
Review: May 2015
