# Supplementary material for: Perioperative blood transfusion is associated with a gene transcription profile characteristic of immunosuppression: a prospective cohort study
Source: Crit Care. 2014 Oct 1;18(5):541. doi: 10.1186/s13054-014-0541-x (PMC4201915; doi:10.1186/s13054-014-0541-x)
Supplement: Additional file 2: — Criteria used for defining the sites of infection. Criteria used for defining the infection sites based on the Center for Disease Control and Prevention definitions [13]. [file 13054_2014_541_MOESM2_ESM.doc]

| **Additional file 2.** Criteria used for defining the sites of infection [13]. | |
| --- | --- |
| **Infection site** | **Definition** |
| **BSI – Bloodsteam Infection**  **(LCBI - Laboratory-Confirmed Bloodstream Infection, Secondary BSI- Secondary Bloodstream Infection)** | **Laboratory-Confirmed Bloodstream Infection (LCBI)** must meet at least 1 of the following criteria:  1. Patient has a recognized pathogen cultured from 1 or more blood cultures.  **AND** organism cultured from blood is not related to an infection at another site.  2. Patient has at least 1 of the following signs or symptoms: fever (>38°C), chills, or hypotension.  **AND** signs and symptoms and positive laboratory results are not related to an infection at another site.  **AND** common skin contaminant is cultured from 2 or more blood cultures drawn on separate occasions.  **Secondary Bloodstream Infection (BSI)**  In a patient suspected of having an infection, blood and a site-specific specimen are collected for culture and both are positive for at least one matching organism. If the site-specific culture is an element used to meet the infection site criterion, then the BSI is considered secondary to that site-specific infection. |

| **Pneumonia** | **Pneumonia** requires any 1 of the following:  1. Rales or dullness to percussion on physical examinations of chest  **AND** any of the following:  A. New onset of purulent sputum or change in character of sputum.  B. Isolation of organism from blood culture.  C. Isolation of pathogen from specimen obtained by trans-tracheal aspirate, bronchial brushing, or biopsy.  2. Chest radiography showing new or progressive infiltrate, consolidation, cavitation, or pleural effusion.  **AND** any of the following:  A. New onset of purulent sputum or change in character of sputum.  B. Isolation of organism from blood culture.  C. Isolation of pathogen from specimen obtained by trans-tracheal aspirate, bronchial brushing, or biopsy.  D. Isolation of virus or detection of viral antigen in respiratory secretions.  E. Diagnostic single antibody titre (IgM) or fourfold increase in paired serum samples (IgG) for pathogen.  F. Histopathologic evidence of pneumonia. |
| --- | --- |

| **Infection site** | **Definition** |
| --- | --- |
| **Wound infection**  **(SIS-Superficial incisional surgical site infection, DIS-Deep incisional surgical site infection)** | **A superficial incisional SSI** must meet the following criterion:  Infection occurs within 30 days after the operative procedure.  **AND** involves only skin and subcutaneous tissue of the incision.  **AND** patient has at least *1* of the following:  A. Purulent drainage from the superficial incision.  B. Organisms isolated from an aseptically obtained culture of fluid or tissue from the superficial incision.  C. At least *1* of the following signs or symptoms of infection: pain or tenderness, localized swelling, redness, or heat, *and* superficial incision is deliberately opened by surgeon and is culture positive or not cultured. A culture-negative finding does not meet this criterion.  D. Diagnosis of superficial incisional SSI by the surgeon or attending physician.  **A deep incisional SSI** must meet the following criterion:  Infection occurs within 30 days after the operative procedure if no implant is left in place.  **AND** involves deep soft tissues (eg, fascial and muscle layers) of the incision.  **AND** patient has at least *1* of the following:  A. Purulent drainage from the deep incision but not from the organ/space component of the surgical site.  B. A deep incision spontaneously dehisces or is deliberately opened by a surgeon and is culture-positive or not cultured when the patient has at least *1* of the following signs or symptoms: fever (>38°C), or localized pain or tenderness. A culture-negative finding does not meet this criterion.  C. An abscess or other evidence of infection involving the deep incision is found on direct examination, during reoperation, or by histopathologic or radiologic examination.  D. Diagnosis of a deep incisional SSI by a surgeon or attending physician. |

| **Urinary tract infection (SUTI)** | **A** **symptomatic urinary tract infection** must meet at least *1* of the following criteria:  1. Patient has at least *1* of the following signs or symptoms with no other recognized cause: fever (>38°C), urgency, frequency, dysuria, or suprapubic tenderness.  **AND** patient has a positive urine culture, that is, ≥10^5^ microorganisms per cc of urine with no more than 2 species of microorganisms.  2. Patient has at least *2* of the following signs or symptoms with no other recognized cause: fever (>38°C), urgency, frequency, dysuria, or suprapubic tenderness.  **AND** at least *1* of the following:  A. Positive dipstick for leukocyte esterase and/or nitrate.  B. Pyuria (urine specimen with ≥10 white blood cell [WBC]/mm^3^ or ≥3 WBC/high-power field of unspun urine).  C. Organisms seen on Gram's stain of unspun urine.  D. At least *2* urine cultures with repeated isolation of the same uropathogen (gram-negative bacteria or *Staphylococcus saprophyticus*) with ≥10^2^ colonies/mL in nonvoided specimens.  E. ≤10^5^ colonies/mL of a single uropathogen (gram-negative bacteria or *S saprophyticus*) in a patient being treated with an effective antimicrobial agent for a urinary tract infection.  F. Physician diagnosis of a urinary tract infection.  G. Physician institutes appropriate therapy for a urinary tract infection. |
| --- | --- |

| **Infection site** | **Definition** |
| --- | --- |
| **Intra-abdominal infection (Organ/space-Organ/space surgical site infection, IAB-Intra-abdominal, not specified elsewhere)** | **Organ/space-Organ/space surgical site infection** must meet the following criterion:  Infection occurs within 30 days after the operative procedure if no implant is left in place or within 1 year if implant is in place and the infection appears to be related to the operative procedure.  **AND** infection involves any part of the body, excluding the skin incision, fascia, or muscle layers, that is opened or manipulated during the operative procedure.  **AND** patient has at least *1* of the following:  A. Purulent drainage from a drain that is placed through a stab wound into the organ/space.  B. Organisms isolated from an aseptically obtained culture of fluid or tissue in the organ/space.  C. An abscess or other evidence of infection involving the organ/space that is found on direct examination, during reoperation, or by histopathologic or radiologic examination.  D. Diagnosis of an organ/space SSI by a surgeon or attending physician.  **IAB-Intra-abdominal, not specified elsewhere** including gallbladder, bile ducts, liver (excluding viral hepatitis), spleen, pancreas, peritoneum, subphrenic or subdiaphragmatic space, or other intraabdominal tissue or area not specified elsewhere must meet at least *1* of the following criteria:  1. Patient has organisms cultured from purulent material from intraabdominal space obtained during a surgical operation or needle aspiration.  2. Patient has abscess or other evidence of intra-abdominal infection seen during a surgical operation or histopathologic examination.  3. Patient has at least *2* of the following signs or symptoms with no other recognized cause: fever (>38°C), nausea, vomiting, abdominal pain, or jaundice.  **AND** at least *1* of the following:  A. Organisms cultured from drainage from surgically placed drain (eg, closed suction drainage system, open drain, T-tube drain).  B. Organisms seen on Gram's stain of drainage or tissue obtained during surgical operation or needle aspiration.  C. Organisms cultured from blood *&* radiographic evidence of infection (eg, abnormal findings on ultrasound, CT scan, MRI, or radiolabel scans [gallium, technetium, etc] or on abdominal x-ray). |

| **Intra vascular catheter related infection (VASC-Arterial or venous infection)** | **Arterial or venous infection** must meet at least 1 of the following criteria:  1. Patient has organisms cultured from arteries or veins removed during a surgical operation.  **AND** blood culture not done or no organisms cultured from blood.  2. Patient has evidence of arterial or venous infection seen during a surgical operation or histopathologic examination.  3. Patient has at least 1 of the following signs or symptoms with no other recognized cause: fever (>38°C), pain, erythema, or heat at involved vascular site.  **AND** more than 15 colonies cultured from intravascular cannula tip using semiquantitative culture method.  **AND** blood culture not done or no organisms cultured from blood.  4. Patient has purulent drainage at involved vascular site.  **AND** blood culture not done or no organisms cultured from blood. |
| --- | --- |

| **Infection site** | **Definition** |
| --- | --- |
| **Skin infection (SKIN)** | **Skin infections** must meet at least *1* of the following criteria:  1. Patient has purulent drainage, pustules, vesicles, or boils.  2. Patient has at least *2* of the following signs or symptoms with no other recognized cause: pain or tenderness, localized swelling, redness, or heat.  **AND** at least *1* of the following:  A. Organisms cultured from aspirate or drainage from affected site; if organisms are normal skin flora they must be a pure culture.  B. Organisms cultured from blood.  C. Positive antigen test performed on infected tissue or blood (eg, herpes simplex, varicella zoster, *H influenzae*, *N meningitidis*).  D. Multinucleated giant cells seen on microscopic examination of affected tissue.  E. Diagnostic single antibody titre (IgM) or 4-fold increase in paired sera (IgG) for pathogen. |
