# Supplementary material for: Perioperative blood transfusion is associated with a gene transcription profile characteristic of immunosuppression: a prospective cohort study
Source: Crit Care. 2014 Oct 1;18(5):541. doi: 10.1186/s13054-014-0541-x (PMC4201915; doi:10.1186/s13054-014-0541-x)
Supplement: Additional file 3: — Multivariable linear regression analysis for prediction of gene expression. Multivariable linear regression analysis of gene expression using requirement for blood transfusion, diagnosis of cancer, duration of the surgical procedure, age, ASA class and the presence of preoperative immunosuppression transfusion as potential independent variables. Selection of variables is described in the Methods section. [file 13054_2014_541_MOESM3_ESM.doc]

| **Additional file 3.** Multivariable Linear Regression Analysis For Prediction of Gene Expression. | | | | | | | | | | | | | | | | |
| --- | --- | --- | --- | --- | --- | --- | --- | --- | --- | --- | --- | --- | --- | --- | --- | --- |
| **Response**  **Variable** | | **Predictor Variables Included in the model** | | | | | | | **Whole**  **Model**  **R^2^** | **Predictors Independently Associated With Response Variable** | | | | | | |
|  |  | Transfusion within 24 hours | Cancer diagnosis | Duration of surgery | Age | ASA class | Sex | Pre-op immune-suppression |  | Transfusion within 24 hours | Cancer diagnosis | Duration of surgery | Age | ASA class | Sex | Pre-op immune-suppression |
| **FOXP3** | ***24HR*** | ***x*** |  |  | ***x*** | ***x*** |  |  | **0.16** | **0.03** |  |  | **0.03** | **0.37** |  |  |
| **FOXP3** | ***48HR*** | ***x*** | ***x*** | ***x*** | ***x*** |  |  |  | **0.16** | **0.05^1^** | **0.45** | **0.15** | **0.15** |  |  |  |
| **GATA3** | ***24HR*** | ***x*** |  |  | ***x*** |  | ***x*** | ***x*** | **0.13** | **0.23** |  |  | **0.03** |  | **0.23** | **0.03** |
| **IL-12** | ***24HR*** | ***x*** |  |  | ***x*** |  |  |  | **0.10** | **0.04** |  |  | **0.04** |  |  |  |
| **TNFα** | ***48HR*** | **x** |  |  | ***x*** |  |  |  | **0.06** | **0.39** |  |  | **0.04** |  |  |  |
| **IL-23** | ***24HR*** | ***x*** |  | ***x*** | ***x*** | ***x*** |  |  | **0.23** | **0.25** |  | **0.0008** | **0.002** | **0.46** |  |  |
| **IL-23** | ***48 HR*** | ***x*** | ***x*** | ***x*** | ***x*** |  |  |  | **0.17** | **0.37** | **0.28** | **0.01** | **0.05** |  |  |  |
| **RORγT** | ***24 HR*** | ***x*** |  |  | ***x*** |  |  |  | **0.15** | **0.04** |  |  | **0.003** |  |  |  |
| **RORγT** | ***48 HR*** | ***x*** | ***x*** |  | ***x*** |  |  |  | **0.19** | **0.05^1^** | **0.34** |  | **0.002** |  |  |  |
| **TNFα / IL-10** | ***24HR*** | ***x*** |  | ***x*** | ***x*** |  |  |  | **0.15** | **0.03** |  | **0.005** | **0.2** |  |  |  |
| **TNFα / IL-10** | ***48HR*** | ***x*** | ***x*** | ***x*** | ***x*** |  |  |  | **0.13** | **0.04^1^** | **0.39** | **0.03** | **0.27** |  |  |  |
| Multivariable linear regression analysis of gene expression using requirement for blood transfusion, diagnosis of cancer, duration of the surgical procedure, age, ASA class and the presence of preoperative immunosuppression transfusion as potential independent variables. Each **x** represents an individual variable included in the model (when the univariate p value <0.1). Selection of variables is described in the methods section. The numbers under ‘*Predictors Independently Associated With Response Variable’* represent the *p-*values obtained when the model was run and are indicative of an independent association with the response variable. *^1^*Indicates *p*-value obtained following backwards elimination of non-significant variables. | | | | | | | | | | | | | | | | |
