# Supplementary material for: Perioperative blood transfusion is associated with a gene transcription profile characteristic of immunosuppression: a prospective cohort study
Source: Crit Care. 2014 Oct 1;18(5):541. doi: 10.1186/s13054-014-0541-x (PMC4201915; doi:10.1186/s13054-014-0541-x)
Supplement: Additional file 4: — Multivariable logistical regression analysis for postoperative infections. Multivariable logistical regression analysis of postoperative infections using cancer diagnosis, duration of surgery and transfusion with the first 24 hours postoperatively as independent variables. Selection of variables is described in the Methods section. [file 13054_2014_541_MOESM4_ESM.doc]

| **Additional file 4.** Multivariable Logistical Regression Analysis for Postoperative Infections | | | | | | | |
| --- | --- | --- | --- | --- | --- | --- | --- |
| **Response**  **Variable** | **Predictor Variables Included in the model** | | | **Whole**  **Model**  **R^2^ (U)** | **Predictors Independently Associated with Response Variable** | | |
|  | Cancer diagnosis | Duration of surgery | Transfusion within 24 hours post-operatively |  | Cancer diagnosis | Duration of surgery | Transfusion within 24 hours post-operatively |
| **Post-operative infectious complications** | ***x*** | ***x*** | ***x*** | **0.10** | **0.006** | **0.08** | **0.007** |
| Multivariable logistical regression analysis of postoperative infections using cancer diagnosis, duration of surgery and transfusion with the first 24 hours postoperatively as independent variables. Each **x** represents an individual variable included in the model (when the univariate p value <0.1) (Table 2). Selection of variables is described in the methods section. The numbers under ‘*Predictors Independently Associated With Response Variable’* represent the *p-*values obtained when the model was run and are indicative of an independent association with the response variable. | | | | | | | |
