# Supplementary material for: Perioperative blood transfusion is associated with a gene transcription profile characteristic of immunosuppression: a prospective cohort study
Source: Crit Care. 2014 Oct 1;18(5):541. doi: 10.1186/s13054-014-0541-x (PMC4201915; doi:10.1186/s13054-014-0541-x)
Supplement: Additional file 5: — A comparison of changes in leucocyte subpopulations with changes in candidate gene expression. Changes in leucocyte subpopulations over the perioperative period are expressed by calculating a ratio of the cell count at 24 hours to the preoperative cell count. The same calculation was used to assess the change in gene expression over the same period. [file 13054_2014_541_MOESM5_ESM.docx]

|  | Neutrophils  24 hours / baseline | Lymphocytes  24 hours / baseline | Monocytes  24 hours / baseline | Eosinophils  24 hours / baseline |
| --- | --- | --- | --- | --- |
| T bet  24 hours/ baseline | r^2^ = 0.0005, p= 0.83 | r^2^ = 0.0005, p=0.82 | r^2^ = 0.02, p=0.21 | r^2^ = 0.0002, p=0.90 |
| TGFβ  24 hours/ baseline | r^2^ = 0.002, p=0.66 | r^2^ = 0.0001, p=0.90 | r^2^ = 0.002, p= 0.65 | r^2^ = 0.0002, p=0.90 |
| TNFα  24 hours/ baseline | r^2^ = 0.001, p=0.72 | r^2^ = 0.002, p=0.62 | r^2^ = 0.002, p=0.68 | r^2^ =0.01, p=0.30 |
| IL-10  24 hours/ baseline | r^2^ = 0.06, p=0.01 | r^2^ = 0.05, p=0.02 (inverse correlation) | r^2^ = 0.006, p=0.44 | r^2^ = 0.01, p=0.29 |
| IL-12  24 hours/ baseline | r^2^ = 0.003, p=0.58 | r^2^ = 0.01, p=0.32 | r^2^ = 0.01, p=0.32 | r^2^ = 0.005, p=0.53 |
| IL-23  24 hours/ baseline | r^2^ = 0.007, p=0.43 | r^2^ = 0.004, p=0.53 | r^2^ = 0.02, p=0.10 | r^2^ = 0.02, p= 0.18 |
| IL- 27  24 hours/ baseline | r^2^ = 0.01, p=0.30 | r^2^ = 0.02, p=0.21 | r^2^ =0.02, p= 0.20 | r^2^ = 0.01, p=0.35 |
| RORγt  24 hours/ baseline | r^2^ = 0.0005, p=0.82 | r^2^ = 0.0001, p=0.91 | r^2^ = 0.03, p= 0.12 | r^2^ = 0.01, p=0.30 |
| Foxp3  24 hours/ baseline | r^2^ = 0.003, p=0.60 | r^2^ = 0.004, p=0.55 | r^2^ = 0.04, p= 0.06 | r^2^ = 0.02, p=0.17 |
| IFNγ  24 hours/ baseline | r^2^ = 0.01, p=0.33 | r^2^ = 0.09, p=0.003 | r^2^ =0.00003, p=0.95 | r^2^ = 0.002, p=0.66 |
| GATA3  24 hours/ baseline | r^2^ = 0.0002, p=0.89 | r^2^ = 0.00001, p=0.99 | r^2^ = 0.03, p=0.08 | r^2^ = 0.01, p= 0.34 |

**Additional file 5:** A comparison of changes in leucocyte subpopulations with changes in candidate gene expression

Changes in leucocyte subpopulations over the perioperative period are expressed by calculating a ratio of the cell count at 24 hours to the preoperative cell count. The same calculation was used to assess the change in gene expression over the same period.
